# Performance characteristics of silicon photomultiplier based 15-cm AFOV TOF PET/CT

**DOI:** 10.1186/s40658-019-0244-0

**Published:** 2019-05-10

**Authors:** Delphine Vandendriessche, Jorge Uribe, Hugo Bertin, Frank De Geeter

**Affiliations:** 1 Department of Nuclear Medicine, Algemeen Ziekenhuis Sint-Jan Brugge-Oostende, Ruddershove 10, 8000 Brugge, Belgium; 2 GE Healthcare, 3000 N Grandview Blvd W-1250, Waukesha, WI 53188 USA; 3 GE Healthcare, Kouterveldstraat 20, 1831 Diegem, Belgium

**Keywords:** Silicon photomultiplier based PET/CT, NEMA, Discovery MI 3-ring, Time-of-flight PET/CT

## Abstract

**Background:**

This paper describes the National Electrical Manufacturers Association (NEMA) system performance of the Discovery MI 3-ring PET/CT (GE Healthcare) installed in Bruges, Belgium. This time-of-flight (TOF) PET camera is based on silicon photomultipliers instead of photomultiplier tubes.

**Methods:**

The NEMA NU2-2012 standard was used to evaluate spatial resolution, sensitivity, image quality (IQ) and count rate curves of the system. Timing and energy resolution were determined.

**Results:**

Full width at half maximum (FWHM) of spatial resolution in radial, tangential and axial direction was 4.69, 4.08 and 4.68 mm at 1 cm; 5.58, 4.64 and 5.83 mm at 10 cm; and 7.53, 5.08 and 5.47 mm at 20 cm from the centre of the field of view (FOV) for the filtered backprojection reconstruction. For non-TOF ordered subset expectation maximization (OSEM) reconstruction without point spread function (PSF) correction, FWHM was 3.87, 3.69 and 4.15 mm at 1 cm; 4.80, 3.81 and 4.87 mm at 10 cm; and 7.38, 4.16 and 3.98 mm at 20 cm. Sensitivity was 7.258 cps/kBq at the centre of the FOV and 7.117 cps/kBq at 10-cm radial offset. Contrast recovery (CR) using the IQ phantom for the TOF OSEM reconstruction without PSF correction was 47.4, 59.3, 67.0 and 77.0% for the 10-, 13-, 17- and 22-mm radioactive spheres and 82.5 and 85.1% for the 28- and 37-mm non-radioactive spheres. Background variability (BV) was 16.4, 12.1, 9.1, 6.6, 5.1 and 3.8% for the 10-, 13-, 17-, 22-, 28- and 37-mm spheres. Lung error was 8.5%. Peak noise equivalent count rate (NECR) was 102.3 kcps at 23.0 kBq/ml with a scatter fraction of 41.2%. Maximum accuracy error was 3.88%. Coincidence timing resolution was 375.6 ps FWHM. Energy resolution was 9.3% FWHM. Q.Clear reconstruction significantly improved CR and reduced BV compared with OSEM.

**Conclusion:**

System sensitivity and NECR are lower and IQ phantom’s BV is higher compared with larger axial FOV (AFOV) scanners like the 4-ring discovery MI, as expected from the smaller solid angle of the 3-ring system. The other NEMA performance parameters are all comparable with those of the larger AFOV scanners.

## Introduction

Over the last years, positron-emission tomography (PET) has benefited enormously from various developments, including time-of-flight (TOF), point spread function (PSF) correction and the introduction of solid-state photomultipliers instead of vacuum photomultiplier tubes [1, 2]. Several manufacturers have now brought solid-state photomultiplier-based systems onto the market with hopes of higher image quality and/or lower radiopharmaceutical dose and improved small lesion detection [3].

In this article, we evaluated the system performance of the Discovery Meaningful Insights PET/CT with a 3-ring PET (Discovery MI 3, GE Healthcare, Milwaukee, WI, USA) and compared it with literature data on the similar system with 4 rings (Discovery MI 4), which had been put onto the market before [3]. The 3-ring configuration provides a 15-cm axial field of view. Each PET-ring is made of 34 units consisting of 4 blocks. Every block has 9 (in the axial direction) × 4 (in the in-plane direction) lutetium-yttrium-oxyorthosilicate crystals, for a total of 14,688 crystals for the system. Each crystal element has a dimension of 3.95 mm (transaxial) × 5.3 mm (axial) × 25 mm (length). Each block is coupled with 6 (in the axial direction) × 3 (in the in-plane direction) silicon photomultiplier arrays (SiPM). The Hamamatsu SiPM array has an active area of 4 × 6 mm and is divided into 2 × 3 pixels. The National Electrical Manufacturers Association (NEMA) NU2-2012 standards [4] were used for the evaluation, as in most of the recent literature [3, 5–17]. Additional measurements were performed according to NEMA NU2-2018 standards [18].

Reconstruction software available on the Discovery MI 3 system includes filtered backprojection (FBP) and Vue Point HD (VPHD), an ordered subset expectation maximization (OSEM) algorithm, which can be combined with PSF correction (VPHD-S). The system also has a TOF ability; Vue Point FX (VPFX) refers to the combination of VPHD with TOF.

With conventional iterative reconstruction algorithms based on maximum likelihood estimation maximization, such as OSEM, quantitative accuracy improves with an increasing number of iterations. To prevent excessive noise propagation, the iterations can be stopped before full convergence, but at the expense of lesser quantitative accuracy. Alternatively, the objective function can be extended with a prior favouring smooth solutions [19] and such algorithms can achieve global convergence while retaining fast initial convergence speed [20]. Q.Clear, a Bayesian penalized likelihood technique, uses a relative difference penalty which is a function of the difference between neighbouring voxels as well as a function of their sum. The penalty acts to suppress noise while preserving edges and is modulated by a penalization factor called beta that can be adapted to the data at hand. In each iteration step, the outcomes with lower variation between neighbouring voxels are favoured over the noisier ones [21–25]. The use of this penalty function thereby allows full convergence, providing more accurate quantitation.

## Materials and methods

NEMA test procedures for Discovery MI were used to perform NEMA tests. Almost all measurements were evaluated per *NEMA Standards Publication NU2-2012* [4]. We made use of the NEMA processing tools contained in the Discovery MI software. Some additional measurements were performed according to NEMA NU2-2018 [18]. Before NEMA testing, a normalization scan and well counter calibration were performed. Additional timing resolution and energy resolution tests were performed.

### Normalization and well counter correction

A normalization scan was performed before the NEMA tests started. We used the calibration/daily quality assurance phantom, which is a 27.6-cm long, 12.5-cm outer diameter (1.3-cm thick) annulus radioactive source filled with ^68^Ge in an epoxy matrix. This phantom is provided with the scanner for calibrations and daily quality assurance. A well counter calibration was performed with 18.38 MBq (at the start of the acquisition) of ^18^F-Fluorodeoxyglucose (^18^F-FDG) in a uniform cylindrical phantom with a diameter of 20 cm and a length of 18 cm. This process provided a normalization sinogram and the activity correction factor.

### Spatial resolution

^18^F-FDG was mixed with a small amount of dye to enhance the visibility of the radioactive liquid. Little drops were suspended on a plate and drawn up by capillary tubes so that the axial length of the drop in the tubes was less than 1 mm. Three point sources were made and inserted in the spatial resolution phantom. The sources were positioned at 1, 10 and 20 cm in the *Y*-direction from the centre of the field of view (FOV). Their positions were adjusted to within ± 1.0 mm of the corresponding nominal positions in the PET’s scan FOV. Data were collected at the centre slice of the FOV and at one eighth from the edge of the axial FOV. Every acquisition consisted of at least 500.000 counts. For NEMA, the images were reconstructed with FBP and VPHD, non-TOF OSEM reconstruction with 34 subsets and 4 iterations without PSF modelling. An additional reconstruction was made using VPHD-S. For each spatial orientation, full width at half maximum (FWHM) and full width at tenth maximum (FWTM) were calculated for every reconstruction and every point source and averaged for the acquisition at the centre of the FOV and at 1/8th axial FOV. FWHM and FWTM were statistically compared between the 3 reconstruction algorithms using correlated sample ANOVA followed by Tukey’s HSD post hoc testing; significance was called at *p* < 0.05. FBP and VPHD were compared on data from the 4-ring systems in Stanford and Uppsala taken from [3] by use of paired *t* tests.

### Sensitivity

A plastic tube (70-cm long and with a lumen of 1 mm) was filled with 16.02 MBq of ^18^F-FDG at time of filling. The activity was left to decay until it was lower than 4 MBq, in order for count losses to be negligible and random coincidences to be low. With the aid of a dedicated source holder and dedicated software, this line source was placed at the centre of the FOV and at a 10-cm radial offset in the *Y*-direction. At each position, 5 1-min scans were made with the number of aluminium sleeves around the plastic tube ranging from 1 to 5. The aluminium ensures the annihilation of all positrons and provides increasing attenuating material. Results were then extrapolated to give the scanner sensitivity with no attenuation material. Data were collected directly from sinograms corrected for randoms. Randoms were subtracted from prompts to obtain trues-only sensitivity results.

### Scatter fraction, count losses and randoms

This test measures the count rate performance of the scanner across a range of radioactivity levels. The scatter fraction portion of this test measures the sensitivity of the scanner to coincidence events caused by scatter.

A 70-cm-long line source with an inner diameter of 3.2 mm containing 851.20 MBq ^18^F-FDG at the start of the acquisition was placed in the NEMA scatter phantom, a 70-cm-long polyethylene cylinder with a diameter of 20 cm. The activity was high enough to achieve count rates beyond the expected peak of the noise equivalent count rate. The phantom was secured from rolling with rubber foam wedges and elevated with a paper stack over the patient table until its centre-line aligned with the scanner’s central axis. The acquisition started with 17 frames of 15 min, without delay between the frames, and ended with 7 frames of 25 min, each with a delay of 25 min. NEMA specifications were used to derive the trues, randoms, scatter and noise-equivalent count rate (NECR) from the prompts dataset in each frame. Randoms were estimated using singles rates and the coincidence timing window that is defined by the manufacturer for clinical use.

### Quantitation accuracy: corrections for count losses and randoms

This test compares the trues rate inferred from count losses and randoms corrections with the trues rate extrapolated from measurements with negligible count losses and randoms. Calculations were done on the data acquired for the test of scatter fraction, count losses and randoms as described above, reconstructed by non-TOF OSEM with 16 subsets and 3 iterations without point-spread function modelling. In each time frame, the absolute value of the error was calculated from a linear fit of the activity concentrations measured below peak NECR using 41 of the 53 slices comprising the phantom volume (the 6 end-slices were ignored); the mean, maximum and minimum error over these 41 slices were derived. The accuracy of the corrections for count losses and randoms was expressed as the maximal absolute value of the error below peak NECR.

### Image quality, attenuation accuracy and scatter correction

The image quality (IQ) test simulates a PET/CT whole body clinical case. The 4 spheres of the IQ phantom with a diameters of 10, 13, 17 and 22 mm were filled with 21 kBq/cc ^18^F-FDG concentration whereas the 2 spheres with a diameter of 28 and 37 mm were filled with water. The background of the phantom was filled with 5.27 kBq/cc ^18^F-FDG, in order to yield a 4:1 concentration ratio between the radioactive spheres and the background volume. The phantom has a cylindrical insert with a diameter of 5 cm, containing a low-density material with an average density of 0.3 g/ml to simulate lung tissue. This insert is positioned in the centre of the phantom to have a non-uniform background. The IQ phantom was centred in the scan FOV. Additional activity (120 MBq) was placed outside the FOV (70-cm-long line source with ^18^F-FDG in the NEMA scatter phantom) to represent scatter radiation. Three acquisitions (with time correction for radioactive decay) were made and reconstructed with the VPFX reconstruction algorithm using a 384 × 384 matrix, CT attenuation correction, 4 iterations, 34 subsets, corrections for randoms, scatter, dead time and normalization. IQ was reported in terms of contrast recovery (CR) and background variability (BV) for the radioactive and non-radioactive spheres and averaged over the three acquisitions for increased reliability. The lung error (LE) is the average of LE from 48 slices out of the 53 slices in the PET image, per [4].

The same acquisitions were reconstructed with the Q.Clear reconstruction algorithm, with a beta value of 50. This low beta value, the same that was used in [3], was selected with the intent of matching the noise levels in the Q.Clear and VPFX images. CR and BV were compared between VPFX and Q.Clear reconstructions by paired *t* tests. For each sphere diameter and reconstruction method, CR and BV were compared amongst the 3-ring system at Bruges and the 4-ring systems at Stanford and Uppsala by calculation of 95% confidence intervals. Significance was called at *p* < 0.05.

An additional acquisition was performed according to NEMA NU2-2018. The 6 spheres of the IQ phantom were now filled with 21.9 kBq/cc ^18^F-FDG concentration, whereas the background was filled with 5.5 kBq/cc ^18^F-FDG concentration, again yielding a 4:1 concentration ratio between the radioactive spheres and the background. Phantom positioning and image reconstruction were identical to those described above for the NEMA NU2-2012 testing. An offline analysis tool was used to derive CR and BV values.

### Timing and energy resolution

Timing resolution was calculated from the acquisition of a line source filled with 16 MBq of ^18^F-FDG and suspended in the centre of the FOV in the axial direction in the smallest aluminium sleeve used in the NEMA sensitivity test. Energy resolution was calculated from an acquisition with a 59 MBq ^68^Ge annular phantom (the scanner’s calibration phantom). Three hundred million counts were taken to acquire the timing spectrum. Measurement of the timing resolution FWHM was based on a 3-point fit of the peak of the timing spectra for each crystal pair after removal of the randoms. The energy spectra were smoothed with a boxcar filter. The timing and energy resolution were calculated for every detector crystal and averaged for the entire system.

### PET/CT alignment

According to NEMA NU2-2018, a PET/CT alignment scan was performed to analyze the registration between the PET and the CT image. An 8-min single-bed-position PET scan was made of the VQC phantom. This phantom consists of 5 point sources of 0.15 MBq ^68^Ge which are visible on both PET and CT images and are embedded in a moulded polyurethane foam. Images were reconstructed using VPFX, in a 256 × 256 matrix, with 16 subsets and 3 iterations and using a standard *Z*-axis filter with 5.0-mm filter cutoff. Dedicated software was used to determine the coordinates of every point source on both PET and CT images. The difference between the PET and CT coordinates along the 3 axes as well as the total distance between the PET and CT positions were calculated for each point source.

### Clinical imaging comparison with Discovery 710 PET/CT

A patient with local recurrence of nasal melanoma was referred to PET for follow-up after chemotherapy and radiation. The patient had a BMI of 24.2 and was injected with 3 MBq/kg for a total of 180 MBq ^18^F-FDG. Ninety minutes after injection, a first TOF acquisition was made on a Discovery 710 PET/CT camera (GE Healthcare, Milwaukee, WI, USA). Two hundred minutes after injection, a second TOF acquisition was made on the Discovery MI 3-ring. The acquisition time at both systems was 13.5 min (1.5 min per bed position). Images were reconstructed using the Q.Clear algorithm, with a beta value of 400 for the Discovery 710 acquisition and 1000 for the Discovery MI 3 acquisition.

## Results

### Spatial resolution

Table 1 summarizes the spatial resolution results for both the FBP and VPHD reconstruction algorithms, as well as for the VPHD-S reconstruction. The results of the NEMA-tests at Stanford and Uppsala with the 4-ring detector, taken from [3] are included in the table for comparison. With FBP, no systematic differences were found between the 3- and 4-ring detector systems, although the tangential resolution on the 3-ring system seemed somewhat better than on the 4-ring system in Stanford and somewhat lower than the 4-ring system in Uppsala. VPHD improved the spatial resolution over that obtained by FBP, although statistical significance was only reached for FWHM in the axial direction and for FWTM in the tangential direction. As expected, VPHD combined with PSF modelling resulted in statistically better resolution than FBP and was also statistically better than VPHD, except for the FWHM radial resolution.

**Table 1:** Spatial resolution

		Filtered backprojection						Non-TOF OSEM (VPHD)						Non-TOF–PSF (VPHD-S)	
		FWHM (mm)			FWTM (mm)			FWHM (mm)			FWTM (mm)			FWHM (mm)	FWTM (mm)
Resolution direction	Radial offset	3R (B)	4R (S)	4R (U)	3R (B)	4R (S)	4R (U)	3R (B)	4R (S)	4R (U)	3R (B)	4R (S)	4R (U)	3R (B)	3R (B)
Radial	1 cm	4.69	4.17	4.02	8.93	9.14	8.52	3.87	3.77	3.67	7.63	7.83	7.74	2.72	5.14
	10 cm	5.58	5.65	5.28	10.31	10.36	9.95	4.8	4.76	4.68	8.92	9.08	9.11	2.81	5.16
	20 cm	7.53	7.52	7.54	13.51	13.88	13.38	7.38	7.36	7.44	13.10	12.99	13.27	2.97	5.58
								NS vs FBP	NS vs FBP	NS vs FBP	NS vs FBP	^*^ vs FBP	NS vs FBP	^*^ vs FBP NS vs VPHD	^*^ vs FBP ^*^ vs VPHD
Tangential	1 cm	4.08	4.40	3.97	8.42	9.17	8.19	3.69	4.00	3.74	7.57	7.95	7.93	2.69	5.10
	10 cm	4.64	4.74	4.23	9.42	9.68	8.83	3.81	4.01	3.82	7.69	8.04	7.86	2.74	5.06
	20 cm	5.08	5.13	4.67	9.69	10.14	9.04	4.16	4.62	4.31	8.11	9.03	8.46	2.71	5.15
								NS vs FBP	^*^ vs FBP	^*^ vs FBP	^*^ vs FBP	^*^ vs FBP	NS vs FBP	^**^ vs FBP ^**^ vs VPHD	^**^ vs FBP ^**^ vs VPHD
Axial	1 cm	4.68	4.57	4.39	10.3	10.38	10.12	4.15	4.00	3.93	9.80	9.80	9.71	3.16	6.71
	10 cm	5.83	6.39	5.63	11.19	12.34	11.8	4.87	5.28	4.3	9.05	8.75	9.34	3.92	7.57
	20 cm	5.47	6.50	5.70	11.14	13.01	12.57	3.98	4.09	4.01	9.55	9.71	9.75	3.07	6.38
								^*^ vs FBP	NS vs FBP	NS vs FBP	NS vs FBP	NS vs FBP	NS vs FBP	^**^ vs FBP ^*^ vs VPHD	^**^ vs FBP ^*^ vs VPHD

3R (B). 4R (S). 4R (U) indicate the three-ring system at Bruges and the four-ring system at Stanford and Uppsala, respectively

Data for 4R (S) and 4R (U) taken from [3]

*TOF* time-of-flight, *PSF* point spread function, *VPHD* VuePoint HD, *VPHD-S* VuePoint HD with PSF correction, *FWHM* full width at half maximum, *FWTM* full width at tenth maximum, *FBP* filtered backprojection

*NS* non-significant; ^*^0.01 ≤ *p* < 0.05; ^**^p < 0.01

### Sensitivity

**Fig. 1:**
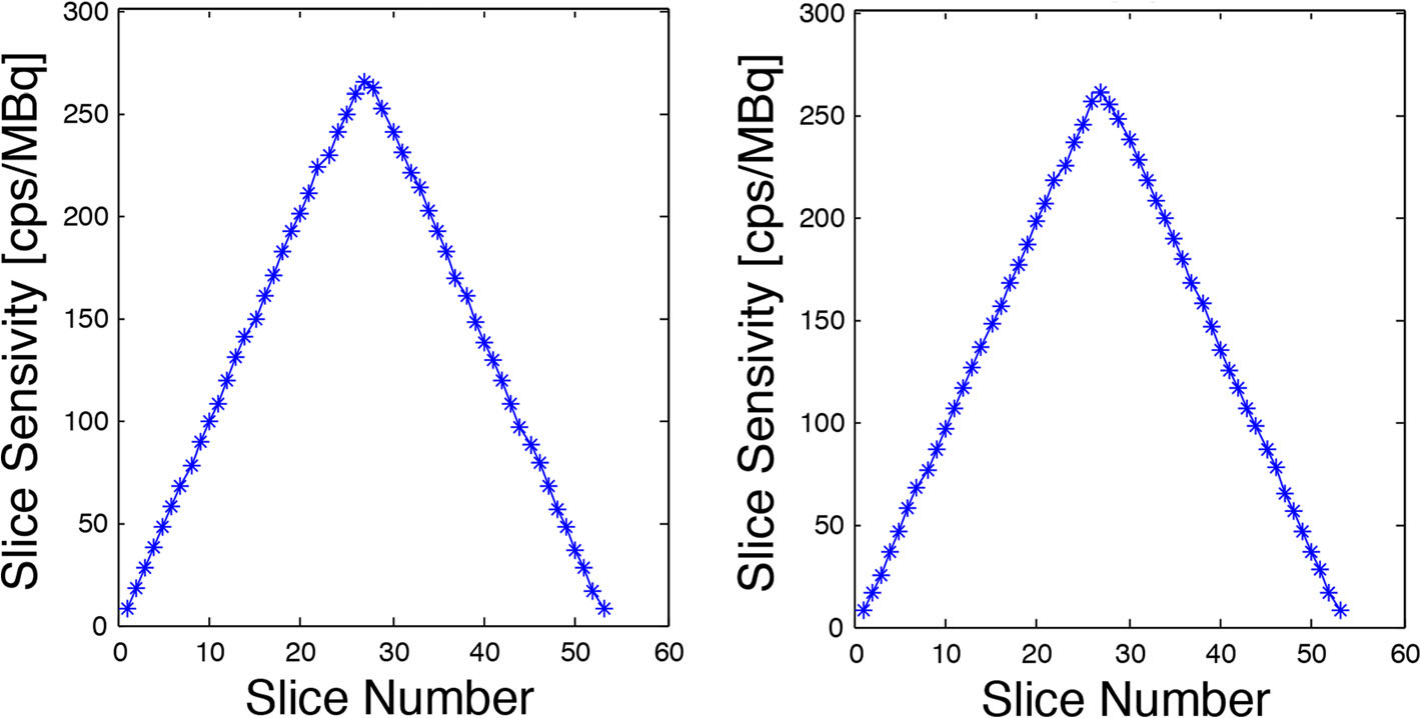
Slice sensitivity profiles. The left panel shows the profile at the centre of the FOV, the right panel is the profile at a 10-cm radial offset in the *Y*-direction. As expected, the sensitivity is less than that of a 20-cm AFOV camera system

The sensitivity at the centre of the FOV was 7.258 cps/kBq. The sensitivity at a radial offset of 10 cm in the Y-direction was 7.117 cps/kBq. Figure 1 shows the slice sensitivity profiles at 0 cm and 10 cm. The sensitivity for the 4-ring system measured at Stanford was 14.0 cps/kBq at the centre of the FOV and 13.8 cps/kBq at a radial offset of 10 cm. As expected, the addition of a fourth ring increases the NEMA sensitivity by the square of the ratio of the axial FOV of the scanners (4/3)^2^.

### Scatter fraction, count losses and randoms

**Fig. 2:**
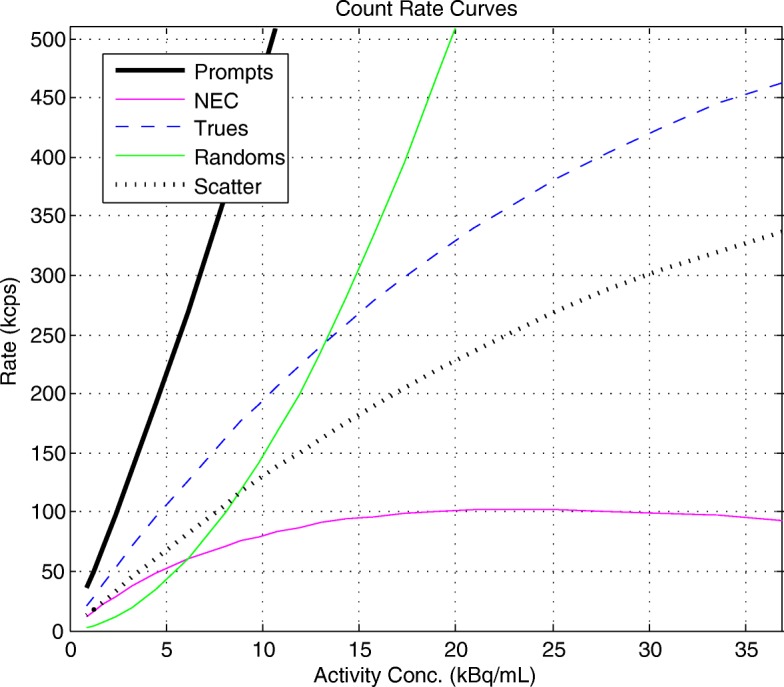
Decay series—count rates as a function of activity concentration. Prompts, trues, randoms, scatters and NECR are depicted as a function of activity concentration. Peak NECR is 102.3 kcps at activity concentration of 23.0 kBq/cc. The peak true counting rate on the MI 3 was 463.1 kcps at 36.9 kBq/ml. NEC noise equivalent counts

**Fig. 3:**
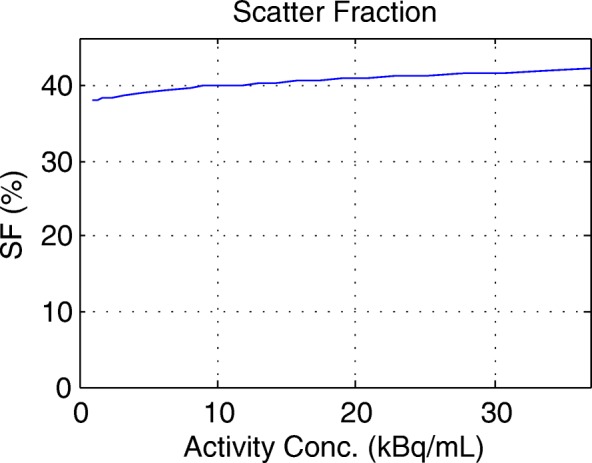
Scatter fraction as a function of activity concentration. Scatter fraction at peak NECR was 41.2% on the Discovery MI 3-ring

Figure 2 shows the total prompts, trues, randoms, scatters and NECR as a function of activity concentration. Figure 3 shows the scatter fraction as a function of activity concentration. Table 2 summarizes the counting rate data and compares them to those on the 4-ring systems at Stanford and Uppsala (data from [3]). Peak NECR was 102.3 kcps; the activity concentration at this peak NECR was 23.0 kBq/cc. Scatter fraction at peak NECR was 41.2%. The peak true counting rate on the MI 3 was 463.1 kcps at 36.9 kBq/ml.

**Table 2:** Counting rate data

Type of measurement	Brugge (this work)	Stanford [3]	Uppsala [3]
Peak NECR (kcps)	102.3	201.1	185.7
Activity at peak NECR (kBq/ml)	23.0	22.1	21.7
Peak true counting rate^a^ (kcps)	463.1	875.9	827.0
Activity at peak true counting rate^a^ (kBq/ml)	36.9	35.4	34.8
Scatter fraction at peak NECR (%)	41.2	40.4	40.8

^a^Note that the experimental setup did not reach the real peak true counting rate, as explained in the text

### Accuracy: correction for count losses and randoms

**Fig. 4:**
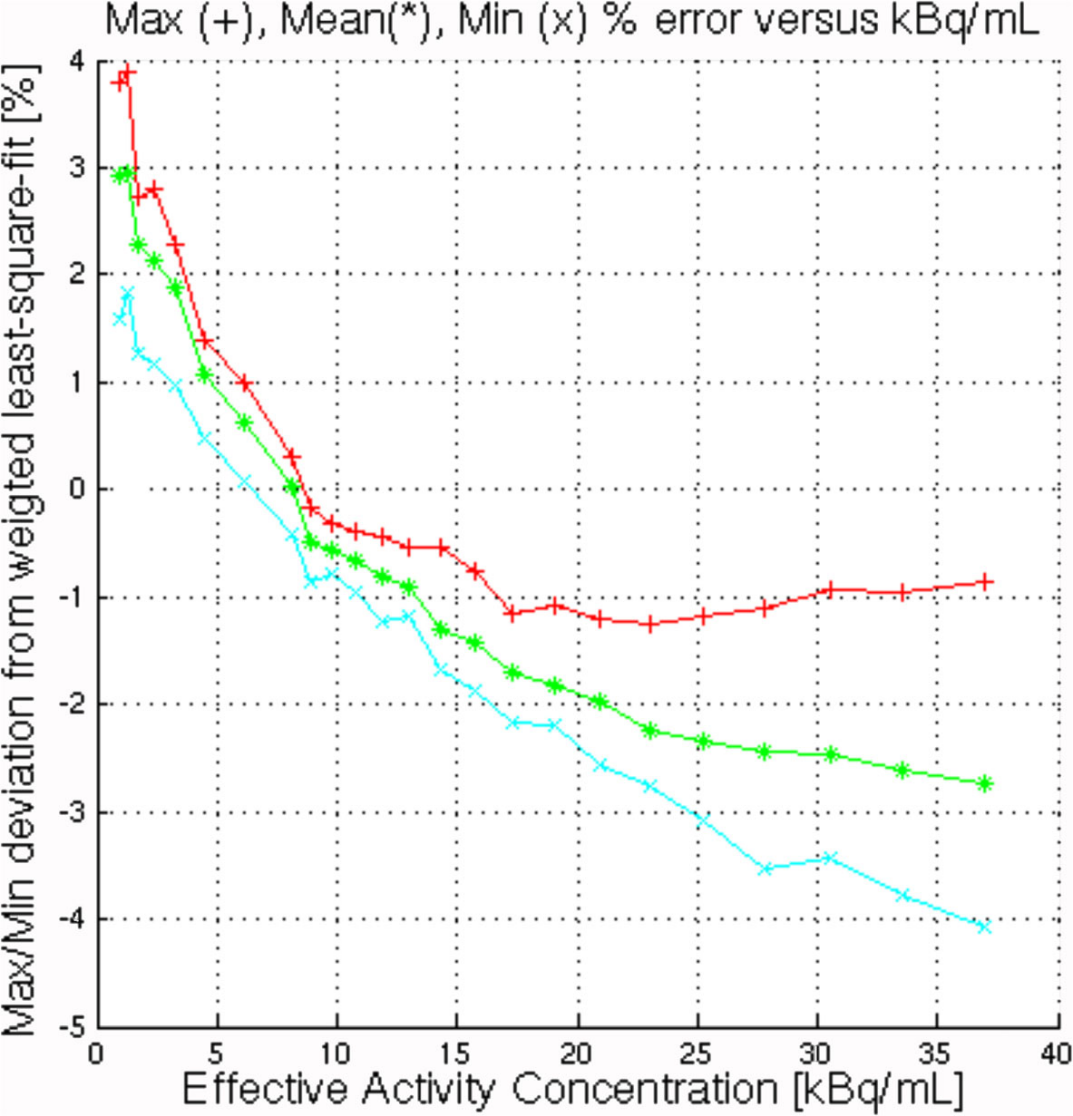
Quantitation accuracy as a function of effective activity concentration. These errors are determined versus least squares fit of quantitation values below peak NECR. Maximal, minimal and mean absolute errors are given over all image slices except the 6 end slices on both ends of the AFOV

Figure 4 shows, as a function of activity concentration, the minimum, maximum and mean error (%) of the measured image quantitation from the expected linear extrapolation from points below peak NECR. Notice that the errors are derived from reconstructed images to which all corrections have been applied, i.e. corrections for attenuation, randoms and scatter. Data points are shown for all activity concentrations probed during the decay series. The maximum deviation from expected activity below peak NECR was 3.88%. For comparison, the maximum deviation on the Discovery MI 4-ring was 2.43% [3].

### Image quality, attenuation accuracy and scatter correction

**Fig. 5:**
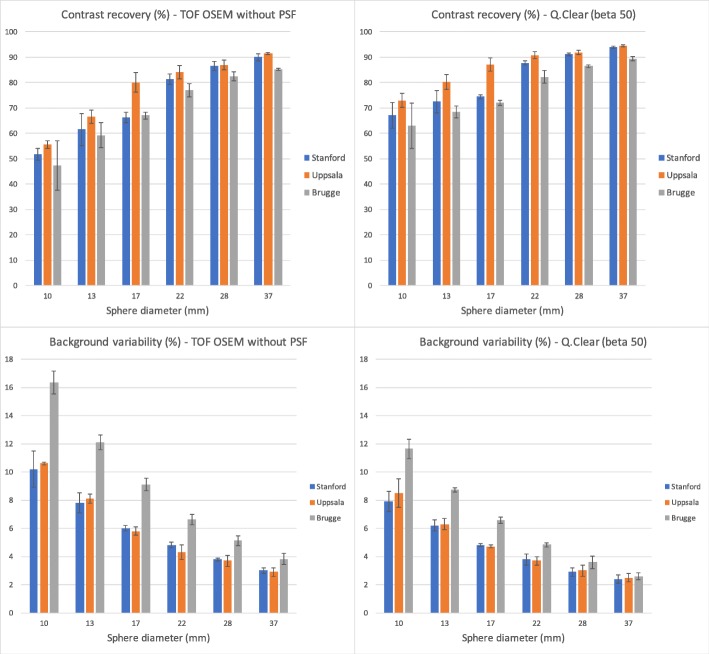
Contrast recovery and background variability. Contrast recovery data are given in the upper panels and background variability in the lower panels. Data are represented for TOF OSEM reconstruction without PSF correction in the left panels and for Q.Clear reconstruction (beta = 50), including PSF correction in the right panels. Data on the MI 4 systems in Stanford and Uppsala are taken from [3]. Error bars represent one standard deviation, as determined from 3 repeat measurements

Figure 5 shows CR (upper panels) and BV (lower panels) in the IQ phantom for the VPFX (TOF OSEM reconstruction without PSF correction) (panels to the left) and for the Q.Clear reconstruction using a beta value of 50 (panels to the right) and compares them with the results for the IQ test on the 4-ring systems in Stanford and Uppsala taken from [3]. Only for the non-radioactive 37-mm sphere, CR with the VPFX reconstruction was statistically lower on the 3-ring system than on the 4-ring systems measured at Stanford and Uppsala. For the 17-mm sphere the value on the 3-ring was similar to that measured on the 4-ring in Stanford, but both were lower than that measured on the 4-ring system in Uppsala. CR was significantly enhanced by use of the Q.Clear reconstruction.

The average lung error for the VPFX reconstruction was 8.5% ± 0.3% and for the Q.Clear reconstruction 5.6% ± 0.2%.

BV with the 3-ring camera was significantly higher for all sphere diameters than for the 4-ring systems. Use of the Q.Clear reconstruction algorithm, however, significantly reduced background variability close to that obtained with the 4-ring system using VPFX reconstruction. This higher background variability observed in the images of the 3-ring scanner compared to the 4-ring scanner is consistent with the shorter acquisition time with which NEMA NU2-2012 penalizes the 3-ring scanner over the 4-ring scanner (3-ring, 3 min 20 s/frame; 4-ring, 4 min 31 s/frame) in order to meet the requirement of scanning 1 m in 30 min.

**Fig. 6:**
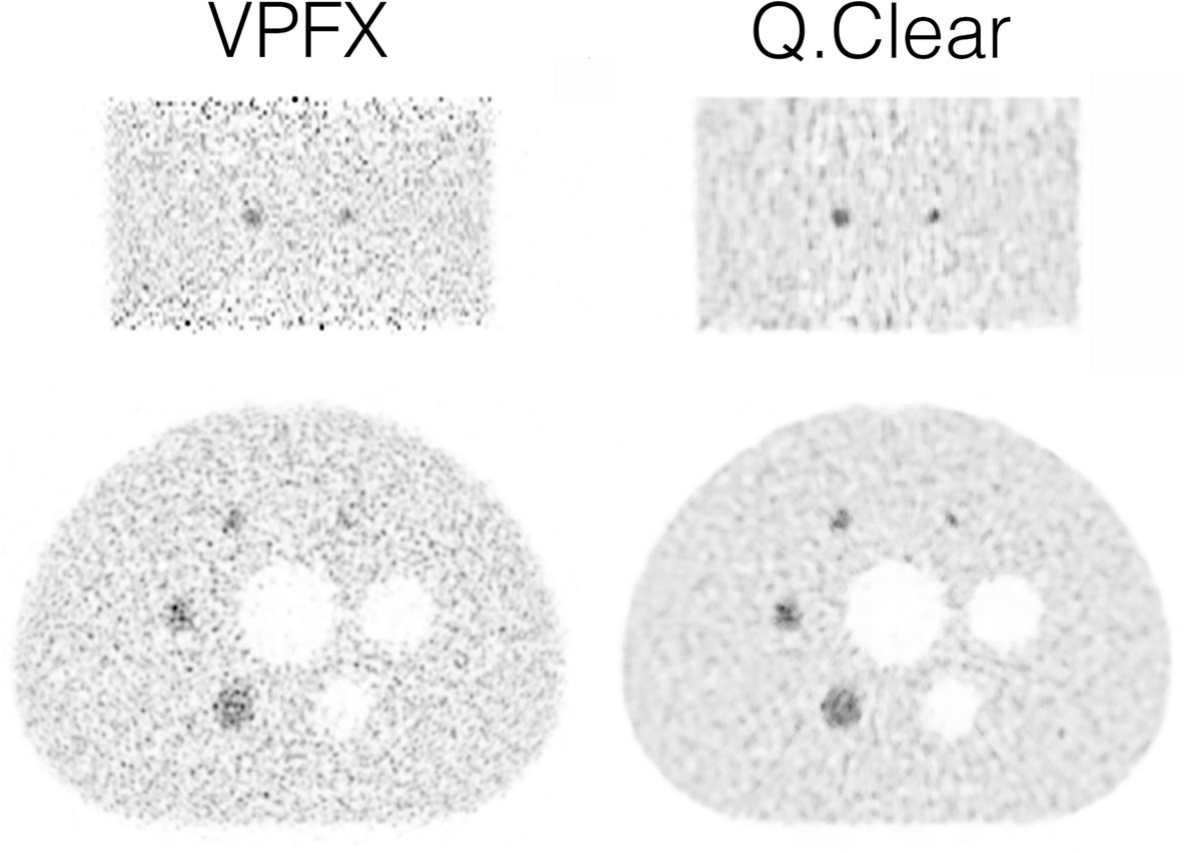
Image quality phantom images. Left images are obtained by VPFX reconstruction, right images by Q.Clear reconstruction with beta = 50. The top row represents coronal slices through 10-mm and 13-mm spheres, the bottom row axial slices through all spheres

The reconstructed axial and coronal images of the NEMA IQ phantom shown in Fig. 6 demonstrate less noise and better CR for the Q.Clear (beta = 50) reconstruction compared with the VPFX reconstruction.

**Fig. 7:**
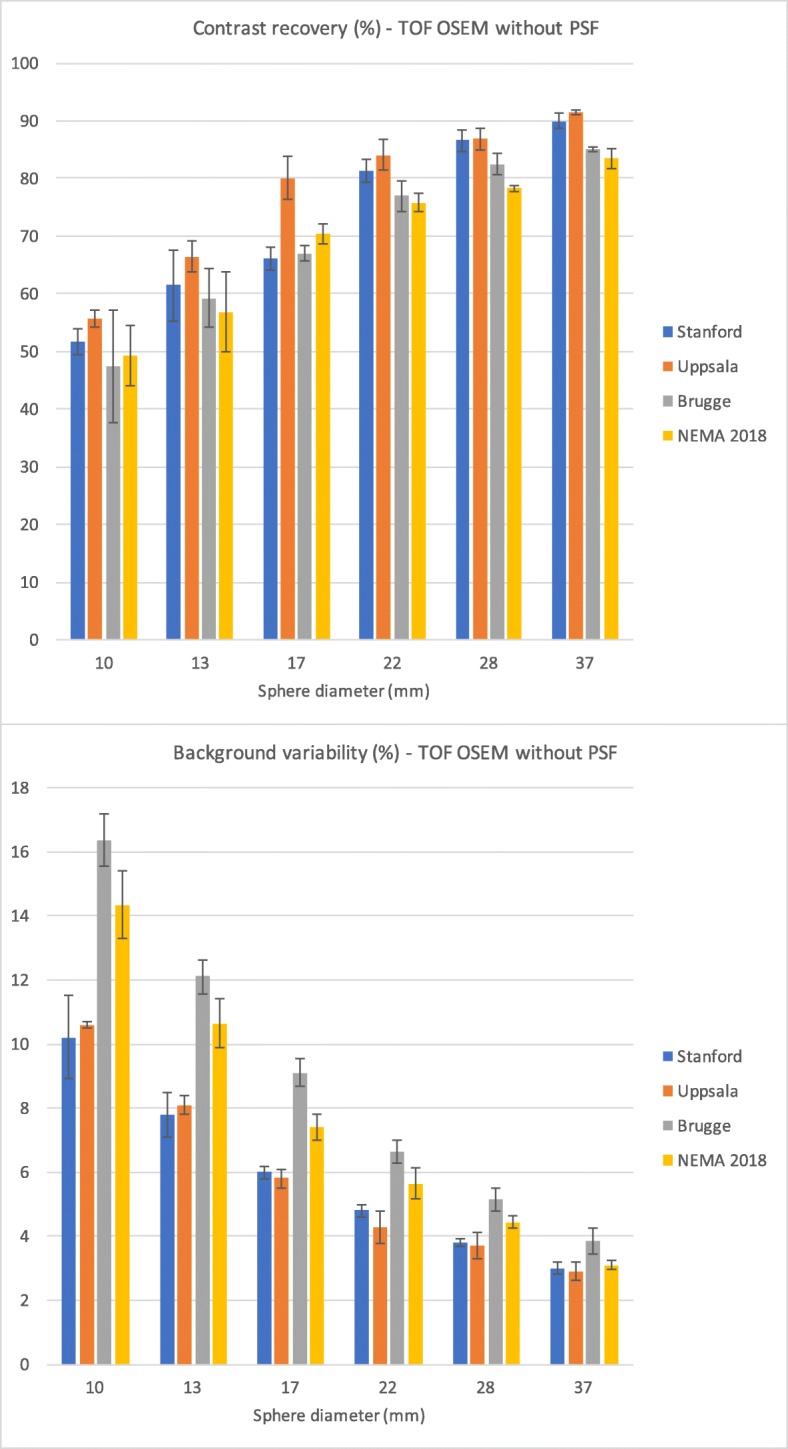
Contrast recovery and background variability according to NEMA NU2-2018. Contrast recovery data are given in the upper panel and background variability in the lower panel. Data are represented for TOF OSEM reconstruction without PSF correction. The data from Fig. 5 are depicted as a reference

Figure 7 shows the IQ data according to NEMA NU2-2018. CR ranged from 49.3% (smallest sphere) to 83.5% (largest sphere). BV ranged from 14.4% (smallest sphere) to 3.1% (largest sphere).

### Timing and energy resolution

The average timing resolution was 375.6 ± 2.7 ps FWHM. Energy resolution was 9.30% ± 0.06% for the 3-ring detector. These results are very close to those obtained on the 4-ring detector [3]: 374.1 ± 2.6 ps FWHM timing resolution and 9.44% ± 0.07% FWHM energy resolution at Stanford and 376.7 ± 2.7 ps and 9.35 ± 0.05% at Uppsala.

### PET/CT alignment

The distance magnitude between the PET and CT position for the five point sources were 1.26 mm, 1.08 mm, 0.55 mm, 0.87 mm and 0.82 mm. The maximal difference between the PET and CT coordinates was 1.03 mm.

### Clinical imaging comparison with Discovery 710 PET/CT

**Fig. 8:**
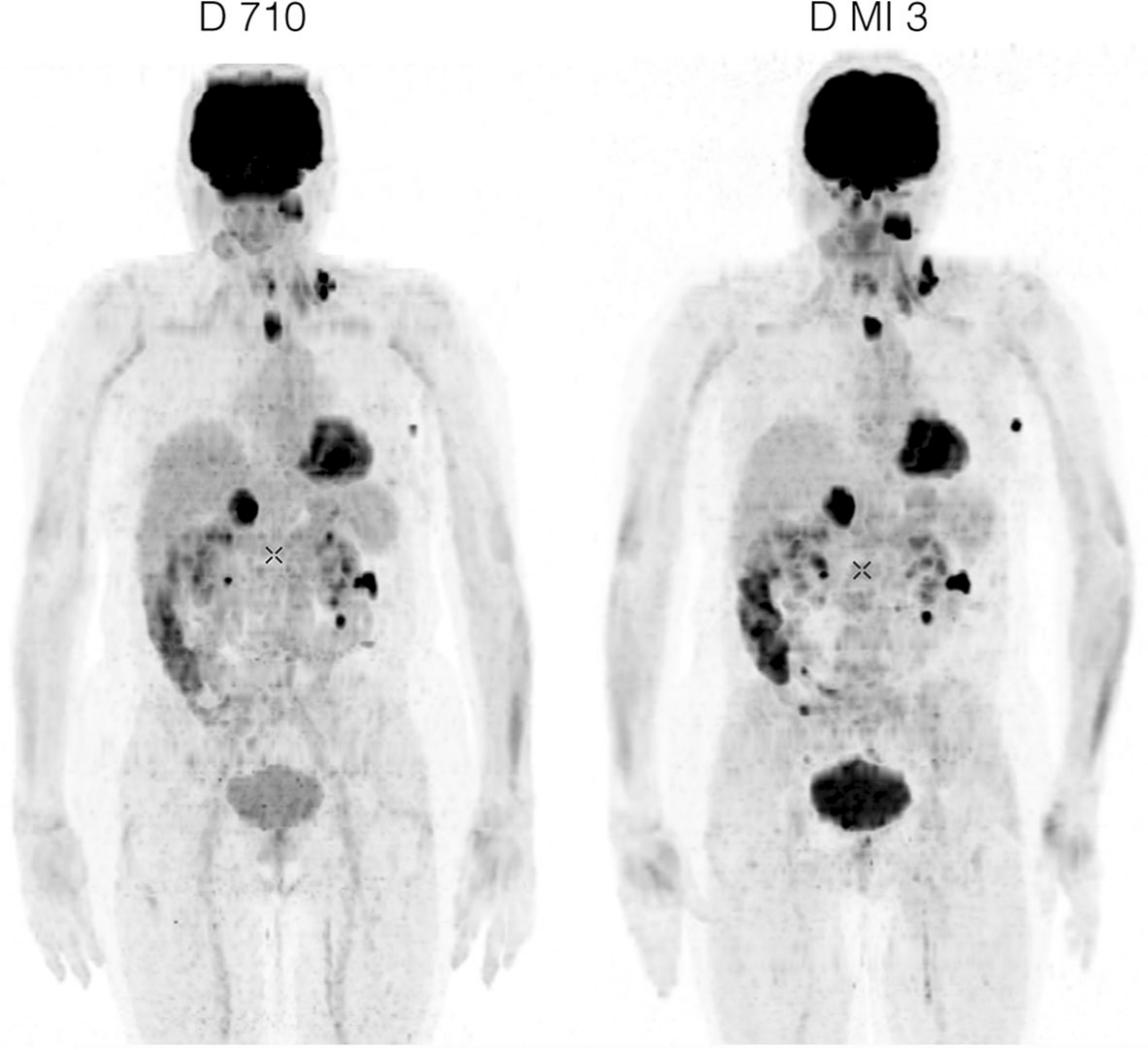
Maximal intensity projection images. These were obtained in a melanoma patient on a Discovery 710 PET/CT (left panel) and, after one half-life radioactive decay, on a Discovery MI 3 PET/CT (right panel), using the same acquisition parameters. Image reconstruction by the Q.Clear algorithm used a beta value of 400 for the Discovery 710 acquisition and 1000 for the MI 3 acquisition. All lesions visible on the Discovery 710 study are visible on the Discovery MI 3 study as well

Figure 8 shows maximal intensity projection images from the Discovery 710 and Discovery MI 3-ring PET/CT, reconstructed with the Q.Clear algorithm using beta values of 400 and 1000, respectively. In spite of one half-life of decay between the two studies, all lesions that were visible on the study performed on the Discovery 710 were also seen on the MI 3-ring study. Biodistribution changed somewhat between the two studies, with vascular activity diminishing and bowel and renal excretory activity increasing. Contrast improved on the later study in a left axillary node.

## Discussion

*Spatial resolution* on our 3-ring system overall was comparable to that on the 4-ring system (Table 1) and other commercially available systems, as can be seen in Table 3. Iterative reconstruction without PSF correction introduces improvement over FBP as further iterating sharpens spatial resolution at the expense of image noise, although it should be acknowledged that the non-negativity constraint in iterative algorithms artificially enhances the apparent spatial resolution [26]. Nonetheless, additional PSF correction accomplished a major improvement due to the better modelling of the detector crystals response.

**Table 3:** NEMA data on PET scanners available on the market

	GE Healthcare				Siemens Healthcare		Philips Healthcare	
Parameter	Discovery MI 3 PET/CT (this work)	Discovery MI 4 PET/CT [3]	Signa PET/MR [14]	Discovery 690/710 PET/CT [6]	Biograph mCT Flow PET/CT [9]	Biograph mMR PET/MR [16]	Vereos Digital PET/CT [17]	Ingenuity TF 128 PET/CT [10]
Axial FOV (cm)	15	20	25	15.7	22.1	25.8	16.4	18
Transverse FOV (cm)	70	70	60	70	70	59.4	67.6	67.6
Detector ring diameter (cm)	74.4	74.4	62.4	81	84.2	65.6	76.4	90
Crystal thickness (mm)	25	25	25	25	20	20	19	22
Spatial resolution FWHM (FBP)								
Radial, 1 cm	4.69	4.1	4.46	4.70^a^	4.33^a^	4.3^a^	4.11^a^	4.84^a^
Tangential, 1 cm	4.08	4.19	4.08	4.70^a^	4.33^a^	4.3^a^	4.11^a^	4.84^a^
Axial, 1 cm	4.68	4.48	5.35	4.74	4.25	4.3	3.96	4.73
Radial, 10 cm	5.58	5.47	5.81	5.34	5.16	5.2	NA	5.25
Tangential, 10 cm	4.64	4.49	4.44	4.79	4.72	4.8	NA	5.01
Axial, 10 cm	5.83	6.01	6.75	5.55	5.85	6.6	NA	5.23
Radial, 20 cm	7.53	7.53	8.42	NA	5.55	NA	5.79	NA
Tangential, 20 cm	5.08	4.9	5.27	NA	6.48	NA	5.79	NA
Axial, 20 cm	5.47	6.1	7.3	NA	7.8	NA	6.2	NA
Sensitivity at centre of FOV (cps/kBq)	7.258	13.7	22.9	7.4	9.6	15	5.7	7.39
Counting rate statistics								
Peak NECR (kcps)	102.3	193.4	214.8	139.1	185	184	171	124.1
Peak NEC activity (kBq/ml)	23	21.9	17.6	29	29	23.1	50	20.3
Peak NEC scatter fraction (%)	41.2	40.6	42.5	37	33.5	37.9	30	36.7
Maximum absolute error (%)	3.88	3.14	3.5	2.09	3.7	5.5	NA	NA
Contrast recovery in spheres								
10 mm	47.4	53.7	36.5	44	28.5	32.5	62	17
13 mm	59.3	64	50.6	56	42.3	50	NA	46
17 mm	67.0	73.1	60	65	58.4	62.9	NA	58
22 mm	77.0	82.7	68.6	75	71.7	70.8	88	63
28 mm	82.5	86.8	80.7	87	70.1	65.1	86	68
37 mm	85.1	90.7	88.6	89	78.3	72.3	89	68
Timing resolution (ps)	375.6	375.4	390	544.3	555^b^	2930	316	502
Energy resolution (%)	9.3	9.4	10.5	12.4	NA	14.5	11.1	11.1

*FOV* field of view, *FWHM* full width at half maximum, *FBP* filtered backprojection, *NA* not applicable, *NEC(R)* noise equivalent count (rate)

^a^Radial and tangential FWHM are averaged

^b^Taken from [3]

*Scanner sensitivity* was close to the value measured on the same system in [27]. It is better than for other systems with comparable FOV: 5.6 cps/kBq for the Biograph mCT Flow with 16.2-cm axial FOV [13] and 5.7 cps/kBq for the Vereos Digital with an axial FOV of 16.4 cm [17] (Table 3). These values are all below those for systems with larger FOV [3, 27], as would be predicted from solid angle considerations, although of course other factors such as detector efficiency play a role as well in determining systems sensitivity. The peak *NECR* of the 3-ring system tested was close to the values measured on the same system in [27]. As expected, it is less than for other systems with larger FOV. The peak true counting rate and the activity at this rate presented in Table 2 were extracted from the decay series at the first acquisition point, which is below the actual concentration needed to reach the trues peak. It was not deemed relevant to expose the operator to the high radiation levels that would be needed to explore a performance parameter irrelevant in clinical operations, considering the high photon sensitivity and low recommended clinical doses. The *quantitation accuracy* below the NEC peak of 3.88% only occurred at activity concentrations that are not clinically relevant.

*Contrast recovery* (Fig. 5) as we measured it on the 3-ring camera was similar to that for the 4-ring system reported by Hsu [3], which reportedly was the best of all systems commercially available (Table 3) [3]. Only for the non-radioactive 37-mm sphere, CR was statistically lower on the 3-ring system than on the 4-ring systems measured at Stanford and Uppsala. For the 17-mm sphere the value on the 3-ring was similar to that measured on the 4-ring in Stanford, but both were lower than that measured on the 4-ring system in Uppsala. As it appears from Fig. 5 (panel A), the latter Uppsala measurement possibly could have been an outlier. Since the background fill (0.6 kBq/ml) in Stanford was lower than in the NEMA specifications (5.3 kBq/ml), another possibility proposed in [3] is that all Stanford values for CR have been somewhat underestimated. Other investigators have also found that CR was similar between systems with a 15-, 20- or 25-cm axial FOV [27]. Except for non-radioactive spheres, CR on the 3-ring system was better than on GE’s Signa PET/MR [14] and on GE’s Discovery 690/710 [6], which already surpass most other systems currently available [3] (Table 3). When a low beta value was used with the purpose of matching the noise level against VPFX reconstructed images, CR was enhanced by the Q.Clear reconstruction algorithm, similar to the 4-ring system [3]. Values on the 3-ring system using Q.Clear were higher than those on the 4-ring system using conventional TOF OSEM without PSF correction.

*Background variability* (Fig. 5) with the 3-ring camera was significantly higher for all sphere diameters than for the 4-ring systems. This has been reported before [27]. Use of the Q.Clear reconstruction algorithm, however, brought back BV close to that obtained with the 4-ring system using OSEM reconstruction.

Neither the *timing resolution* of 375.6 ps nor the *energy resolution* of 9.3% was different from those on the 4-ring system.

*Registration of the PET and CT images* was excellent with the maximal distance between the CT and PET coordinates being less than 1.03 mm for all point sources along all axes.

The improved CR for small lesions and the increased timing resolution explain why the 3-ring Discovery MI was shown (Fig. 8) to afford at least equal IQ on half of the radioactivity dose as compared with a similar acquisition on a Discovery 710 PET camera with conventional detectors. Increased image quality, diagnostic confidence and accuracy with digital PET cameras have been reported before [3, 28]. On a Discovery MI-4 ring system, acquisition times as fast as 90 s per bed position have been demonstrated to result in acceptable image quality, even after long delays from injection to imaging [29]. In the latter study, image quality was rated by two experienced nuclear physicians on a 5-point Likert scale (non-diagnostic, sub-optimal, acceptable, good, excellent) and supported by measurement of the standardized uptake value of a representative lesion and of the signal-to-noise ratio in the liver. Short acquisitions not only may increase patient throughput. They may also be critical to avoid patient movement artefacts in those patients unable to remain still for longer periods of time or to minimize the time of sedation or anaesthesia in patients requiring this, e.g. paediatric patients. The implications of the alternative possibility of injecting less radioactivity on patient and personnel dosimetry as well as on tracer cost are evident. The issue of patient dosimetry is all the more important in those patients with a high life expectancy, e.g. paediatric patients [30, 31], or those who will require repeat studies over the course of their disease, e.g. lymphoma patients [32].

## Conclusions

As expected from the smaller solid angle of the 3-ring camera, scanner sensitivity and NECR are lower and background variability is higher than those on the 4-ring digital camera system. Other NEMA specifications on the 3-ring digital PET/CT camera are all comparable to those on the 4-ring digital camera system. The SiPM-based PET may provide equal image quality within half of the acquisition time or with half the amount of tracer injected compared with a PET system based on vacuum photomultiplier tubes. Q.Clear reconstruction of the NEMA-IQ phantom with low beta values improves contrast recovery and diminishes background variability, when compared to images reconstructed with the manufacturer’s recommended OSEM protocol.

## Abbreviations

AFOV: Axial field of view
BV: Background variability
CR: Contrast recovery
CT: Computed tomography
FBP: Filtered backprojection
FDG: Fluorodeoxyglucose
FOV: Field of view
FWHM: Full width at half maximum
FWTM: Full width at tenth maximum
GE: General Electric
IQ: Image quality
LE: Lung error
MR(I): Magnetic resonance (imaging)
NEC(R): Noise equivalent count (rate)
NEMA: National Electrical Manufacturers Association
OSEM: Ordered subset expectation maximization
PET: Positron-emission tomography
PSF: Point spread function
SiPM: Silicon photomultiplier
TOF: Time-of-flight
VPFX: Vue Point FX (= VPHD + TOF)
VPHD: Vue Point HD (= OSEM)
